# Association between residual cholesterol and sarcopenia in American adults

**DOI:** 10.3389/fendo.2024.1461961

**Published:** 2024-11-28

**Authors:** Jianzhao Li, Yuning Lin

**Affiliations:** Department of Orthopaedics, The first people’s hospital of Zhaoqing, Zhaoqing, China

**Keywords:** remnant cholesterol, lipid, cholesterol, sarcopenia, NHANES

## Abstract

**Background:**

Remnant cholesterol (RC) is a novel lipid metabolism indicator; however, its relationship with sarcopenia has not been clearly established. This study was conducted to explore the association between RC and sarcopenia.

**Methods:**

An analysis was performed utilizing cross-sectional data from the NHANES 2011–2018. The variable RC was subjected to a logarithmic transformation to address its skewness. Logistic regression studies were conducted to examine the association between RC and sarcopenia. This study used restricted cubic spline (RCS) and threshold saturation techniques to investigate nonlinear connections. Subgroup, sensitivity, and additional analyses were performed to assess the robustness and validity of the findings.

**Results:**

The study included 4636 participants. Participants with sarcopenia had significantly higher RC levels. Logistic regression demonstrated a substantial positive association between the prevalence of sarcopenia and log RC (OR=1.69, 95% CI=1.32-2.17). RCS analysis revealed a nonlinear relationship, identifying a threshold at RC=25. When the RC is below this threshold, every one-unit increase in RC increases the chance of sarcopenia by 7% (OR=1.07, 95% CI=1.04-1.10); above this threshold, changes in RC were not significant. Subgroup analysis confirmed that RC was an independent risk factor for sarcopenia. The sensitivity and supplementary analyses supported the main findings.

**Conclusion:**

This study demonstrates a significant positive association between RC levels and the prevalence of sarcopenia in U.S. adults, offering novel evidence that RC may serve as a valuable indicator for sarcopenia assessment.

## 1 Introduction

The progressive loss of skeletal muscle mass and strength is the hallmark of sarcopenia, which typically results in a decline in physical function and a lower quality of life (1, 2). It is significantly linked to functional impairment, disability, and increased mortality, and it increases the risk of accidents and fractures (3, 4). Although primarily associated with aging, sarcopenia has also been reported in younger populations (5). Studies indicate that sarcopenia affects 10–16% of elderly individuals worldwide (3). It is anticipated that the prevalence of sarcopenia will increase as the global population ages, which could result in a substantial increase in healthcare expenses and present substantial obstacles for public health systems. Recently, an increasing number of studies have focused on sarcopenia, and identifying the factors that contribute to the prevalence of sarcopenia is critical to improving health and quality of life (6–8).

Recently, remnant cholesterol (RC) has gained attention as a novel indicator of lipid metabolism (9). Traditional lipid testing primarily focuses on low-density lipoprotein cholesterol (LDL-C) and high-density lipoprotein cholesterol (HDL-C). RC is a triglyceride-rich lipoprotein, and a number of previous studies have confirmed the strong association between RC and health conditions (10–12). One study indicated that RC is negatively correlated with total spine bone mineral density (BMD) in American adults, highlighting the importance of RC in bone health (13). Similarly, another study showed a significant positive association between RC values and hypertension, type 2 diabetes, and their coexisting risks, and may be mediated by inflammatory responses (14). Previous studies have shown that elevated RC levels are strongly associated with chronic inflammation and oxidative stress, which play an important role in the development of sarcopenia (14–17). However, there is a lack of current systematic research on the association of RC with sarcopenia, and filling this gap could help to understand the potential role of RC in muscle health. Assuming that RC is positively associated with the prevalence of sarcopenia, this finding will help expand the field of sarcopenia research and provide new indicators for identifying populations at high risk for sarcopenia.

## 2 Methods

### 2.1 Survey description

The National Health and Nutrition Examination Survey (NHANES) is a cross-sectional study that assesses the health and nutritional status of the U.S. population through the use of a complex stratified multistage sampling design with data covering physical examination results, laboratory data, and questionnaires on lifestyle and nutritional status. To guarantee the accuracy and representativeness of the sample, this investigation implements a multistage probability sampling methodology. The ethics review board approved the study, and all participants provided written informed consent. NHANES was recently used for sarcopenia evaluation in different studies (18–20).

### 2.2 Study population

Data from the NHANES 2011–2018 cycles, which span four cycles, were used in this study. The following criteria were used to determine inclusion: (1) participants were over 20 years old and pregnant, (2) participants had full data on sarcopenia, and (3) participants had complete data on RC. As the sarcopenia measurement data covered participants aged between 8 and 59 years, the final age range of the sample included in this study after passing the screening criteria was 20-59 years, an age range that makes the findings more generalizable and population-specific.

### 2.3 Calculation of RC

The RC concentration was calculated using a formula from previous studies that incorporated anthropometric data and blood biomarkers. The formula used was RC (mg/dL) = TC (mg/dL) - HDL (mg/dL) - LDL (mg/dL).

### 2.4 Definition of sarcopenia

Different definitions of sarcopenia exist. We used one from the Osteoarthritis Research Society International (OARSI), which defines sarcopenia as having a sarcopenia index (SMI) below 0.789 for males and 0.512 for females, according to their standards (21, 22). In NHANES, dual-energy X-ray absorptiometry (DEXA) is used to measure attached skeletal muscle mass (ASM). where ASM did not include bone mineral content (BMC) to avoid interference of BMD in the assessment of sarcopenia. The SMI was calculated as the ratio of total ASM (kg) to BMI (kg/m²).

### 2.5 Covariates

This study considered various covariates, including demographic characteristics, lifestyle factors, health status, and dietary habits. The demographic characteristics included age, sex, race, poverty index ratio (PIR), and education level. Lifestyle factors included smoking behavior and physical activity level. Smoking behavior was determined by a questionnaire, defining smokers as those who had smoked more than 100 cigarettes during their lifetime. Metabolic equivalents (MET) were employed to determine physical activity levels, and individuals with MET values below 600 (minutes/week) were classified as inactive. Health status was determined by physician diagnoses or self-reports, including diabetes, hypertension, hypercholesterolemia, and chronic kidney disease. Dietary habits included daily intake of energy, protein, carbohydrates, total sugar, dietary fiber, and total fat.

### 2.6 Statistical analysis

This study used data from the NHANES database from 2011 to 2018, covering four survey cycles, and to ensure that the sample was nationally representative, a weighted approach was used to correct for sampling bias by adjusting the data according to the sample weights provided by NHANES (WTMEC2YR). Data processing included the exclusion of missing data for the primary exposure variable and the outcome variable, whereas missing data for covariates were retained to maximize sample size. The baseline characteristics of the final participants were categorized by sarcopenia status in the descriptive analysis. The RC variable was log-transformed to reduce the effect of skewness and to bring it closer to a normal distribution. Logistic regression analysis was used to investigate the association between RC and sarcopenia, while accounting for numerous variables. The robustness of the results was improved by dividing RC into four categories to further investigate the association trends between sarcopenia and various RC levels. The study used restricted cubic spline (RCS) analysis to ascertain the nonlinear association between RC and sarcopenia. Additionally, threshold effect analysis was utilized to identify the crucial points. Subgroup analyses were conducted to examine the possible influence of stratification variables on the relationship between RC and sarcopenia. Ultimately, sensitivity and supplemental analyses were performed to assess the strength and reliability of the findings. The analyses were performed using R software (version 4.2.3), and statistical significance was defined as P < 0.05.

## 3 Results

### 3.1 Baseline characteristics

**Figure 1:**
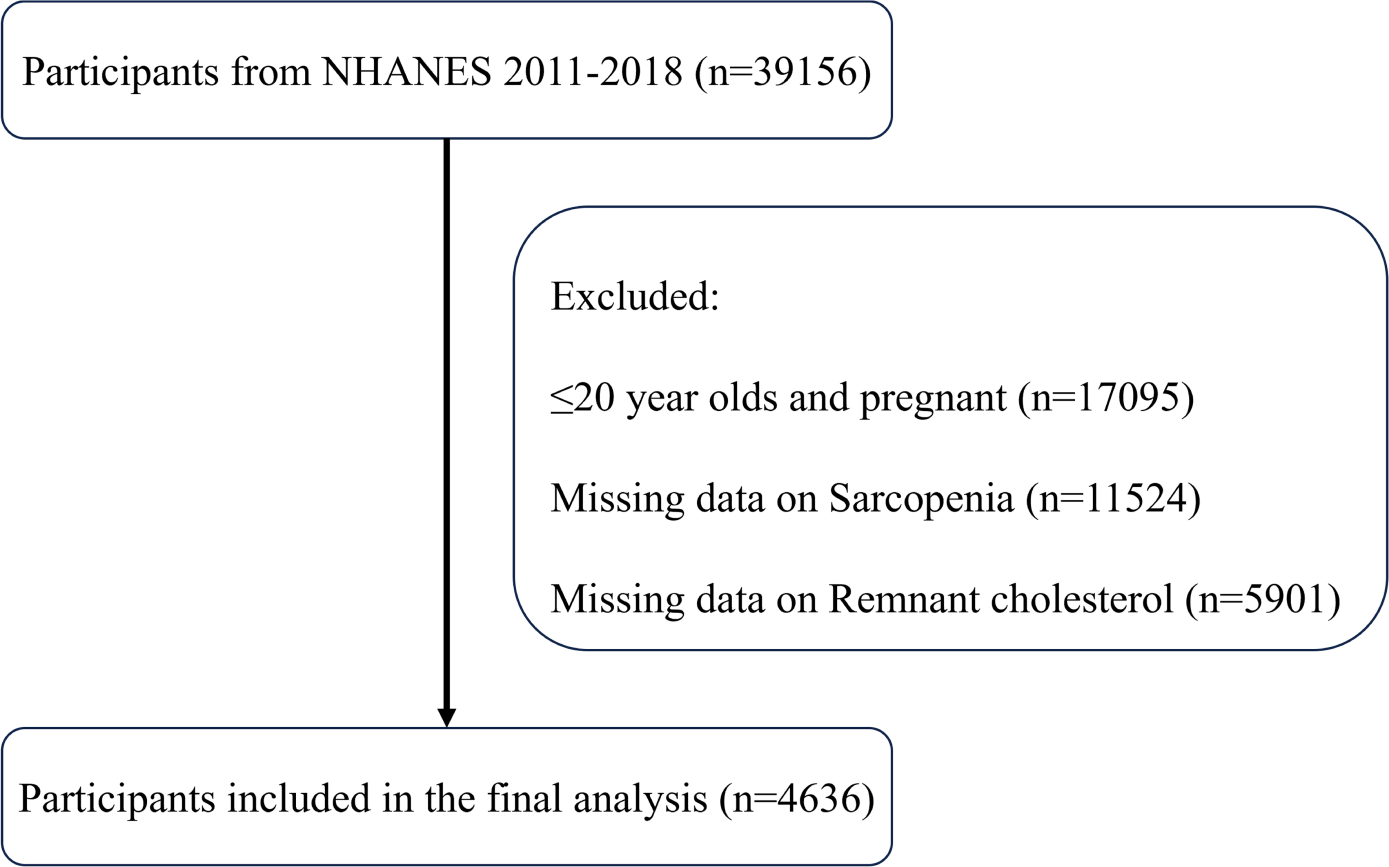
Protocol of including participants.

As shown in **Figure 1**, this study initially included all participants in the NHANES data from 2011 to 2018, totaling 39,156 participants. First, 17,095 participants in the ≤20-year-old and pregnant populations were excluded, and next, 11,524 participants with missing sarcopenia measurements and 5,901 participants with missing RC data were excluded, respectively. Ultimately, the study sample included 4636 participants who met the inclusion criteria for this analysis. **Table 1** displays the initial characteristics of the individuals categorized by sarcopenia status. The study included 4221 non-sarcopenia and 415 sarcopenia participants. Participants with sarcopenia were generally older, had lower education levels and incomes and were more likely to be of Mexican descent. These participants had less physical activity, higher BMI and total cholesterol levels, and lower intakes of energy, protein, carbohydrates, and total fat. Additionally, individuals with sarcopenia were more likely to have diabetes, coronary heart disease, chronic kidney disease, hypertension, and hypercholesterolemia. Notably, these participants had higher RC levels, suggesting a potential link between RC and sarcopenia.

**Table 1:** Baseline characteristics of the study population.

Characteristic	Overall	Non-Sarcopenia	Sarcopenia	P-value
n	4636	4221	415	
Age (%)				<0.001
<40	2377 (51.3)	2237 (53.0)	140 (33.7)	
>40	2259 (48.7)	1984 (47.0)	275 (66.3)	
Sex (%)				0.781
Female	2366 (51.0)	2151 (51.0)	215 (51.8)	
Male	2270 (49.0)	2070 (49.0)	200 (48.2)	
Race (%)				<0.001
Mexican American	683 (14.7)	546 (12.9)	137 (33.0)	
Non-Hispanic Black	930 (20.1)	908 (21.5)	22 (5.3)	
Non-Hispanic White	1632 (35.2)	1513 (35.8)	119 (28.7)	
Others	1391 (30.0)	1254 (29.7)	137 (33.0)	
Education level (%)				<0.001
Under high school	872 (18.8)	730 (17.3)	142 (34.2)	
High school	991 (21.4)	894 (21.2)	97 (23.4)	
Above high school	2772 (59.8)	2596 (61.5)	176 (42.4)	
No record	1 (0.0)	1 (0.0)	0 (0.0)	
PIR (%)				<0.001
<1	930 (22.0)	826 (21.4)	104 (28.1)	
1-3	1728 (40.9)	1566 (40.6)	162 (43.8)	
>3	1572 (37.2)	1468 (38.0)	104 (28.1)	
Activity status (%)				<0.001
Active	2844 (61.3)	2645 (62.7)	199 (48.0)	
Inactive	1792 (38.7)	1576 (37.3)	216 (52.0)	
Smoke (%)				0.869
No	2772 (59.8)	2521 (59.7)	251 (60.5)	
Yes	1862 (40.2)	1698 (40.2)	164 (39.5)	
No record	2 (0.0)	2 (0.0)	0 (0.0)	
Diabate (%)				<0.001
No	4201 (90.6)	3863 (91.5)	338 (81.4)	
Yes	343 (7.4)	281 (6.7)	62 (14.9)	
No record	92 (2.0)	77 (1.8)	15 (3.6)	
CAD (%)				0.022
No	4527 (97.6)	4129 (97.8)	398 (95.9)	
Yes	109 (2.4)	92 (2.2)	17 (4.1)	
CKD (%)				<0.001
No	4541 (98.0)	4146 (98.2)	395 (95.2)	
Yes	93 (2.0)	73 (1.7)	20 (4.8)	
No record	2 (0.0)	2 (0.0)	0 (0.0)	
Hypertension (%)				<0.001
No	3507 (75.6)	3230 (76.5)	277 (66.7)	
Yes	1123 (24.2)	986 (23.4)	137 (33.0)	
No record	6 (0.1)	5 (0.1)	1 (0.2)	
Hypercholesterolemia (%)				<0.001
No	3482 (75.1)	3216 (76.2)	266 (64.1)	
Yes	1141 (24.6)	996 (23.6)	145 (34.9)	
No record	13 (0.3)	9 (0.2)	4 (1.0)	
HDL (mean (SD)) (mg/dL)	53.20 (15.10)	53.58 (15.20)	49.41 (13.46)	<0.001
LDL (mean (SD)) (mg/dL)	114.24 (34.36)	113.59 (34.32)	120.85 (34.13)	<0.001
TC (mean (SD)) (mg/dL)	189.57 (38.84)	188.81 (38.71)	197.32 (39.34)	<0.001
RC (mean (SD)) (mg/dL)	22.12 (13.49)	21.64 (13.39)	27.07 (13.56)	<0.001
BMI (mean (SD)) (kg/m^2^)	28.75 (6.85)	28.26 (6.55)	33.76 (7.72)	<0.001
ASM (mean (SD)) (kg)	22.59 (6.34)	22.82 (6.31)	20.22 (6.13)	<0.001
SMI (mean (SD))	0.80 (0.20)	0.82 (0.20)	0.60 (0.13)	<0.001
Energy (mean (SD)) (kcal)	2108.34 (821.97)	2125.61 (827.06)	1932.81 (747.07)	<0.001
Protein (mean (SD)) (g)	83.46 (36.14)	84.17 (36.35)	76.22 (33.05)	<0.001
Carbohydrate (mean (SD)) (g)	253.89 (106.06)	255.24 (106.67)	240.09 (98.79)	0.011
Total sugars (mean (SD)) (g)	108.12 (63.71)	108.61 (63.66)	103.17 (64.08)	0.129
Dietary fiber (mean (SD)) (g)	17.36 (9.61)	17.41 (9.66)	16.82 (9.10)	0.277
Total fat (mean (SD)) (g)	80.80 (37.82)	81.58 (38.09)	72.80 (34.01)	<0.001

Mean (SD) for continuous variables, % for categorical variables. ASM, appendicular skeletal muscle; BMI, body mass index; CAD, coronary artery disease; CKD, chronic kidney disease; HDL, high density lipoprotein; LDL, low density lipoprotein; TC, Total cholesterol; RC, remnant cholesterol; WC, waist circumference.

### 3.2 Association between RC and sarcopenia prevalence

Logarithmic transformation of RC was performed to correct the left-skewed data. The logistic regression analysis of the relationship between RC and sarcopenia incidence is illustrated in **Table 2**. According to Model 1, the incidence of sarcopenia was significantly positively correlated with the log RC (OR=2.32, 95% CI=1.92-2.80). After accounting for several factors, Model 3 demonstrated that for each unit increase in log RC, the occurrence of sarcopenia increased by 69% (OR=1.69, 95% CI=1.32-2.17). After converting RC into four categorical variables, the relationship between RC levels and sarcopenia was studied. Compared with the lowest quartile, an increase in the incidence of sarcopenia was significantly correlated with higher RC levels (P for trend<0.001). Even after adjusting for all covariates, this trend remained significant for the highest RC level (OR=2.60, 95% CI=1.61-4.20), indicating a robust positive association between RC and sarcopenia.

**Table 2:** The relationship between RC and Sarcopenia.

		Model 1 OR (95%CI) P-value	Model 2 OR (95%CI) P-value	Model 3 OR (95%CI) P-value
Sarcopenia	log RC	2.32 (1.92, 2.80) <0.001	2.04 (1.68, 2.48) <0.001	1.69 (1.32, 2.17) <0.001
	Q1	[Reference]	[Reference]	[Reference]
	Q2	2.54 (1.54, 4.20) <0.001	2.28 (1.38, 3.76) 0.002	2.01 (1.14, 3.57) 0.018
	Q3	4.26 (2.96, 6.13) <0.001	3.48 (2.41, 5.04) <0.001	2.99 (1.80, 4.94) <0.001
	Q4	4.68 (3.17, 6.91) <0.001	3.64 (2.49, 5.31) <0.001	2.60 (1.61, 4.20) <0.001
	P for trend	<0.001	<0.001	0.004

CI, confidence Interval; OR, odds ratio; Q, quartiles; RC, remnant cholesterol.

Model 1: no covariates adjusted; Model 2: adjusted for age, sex, and race; Model 3: adjusted for age, sex, race, educational level, PIR, smoke, activity status, hypertension, hypercholesterolemia, CAD, CKD, diabetes, energy, protein, carbohydrate, total sugars, dietary fiber, total fat.

### 3.3 Nonlinear relationship and threshold effect analysis

**Figure 2:**
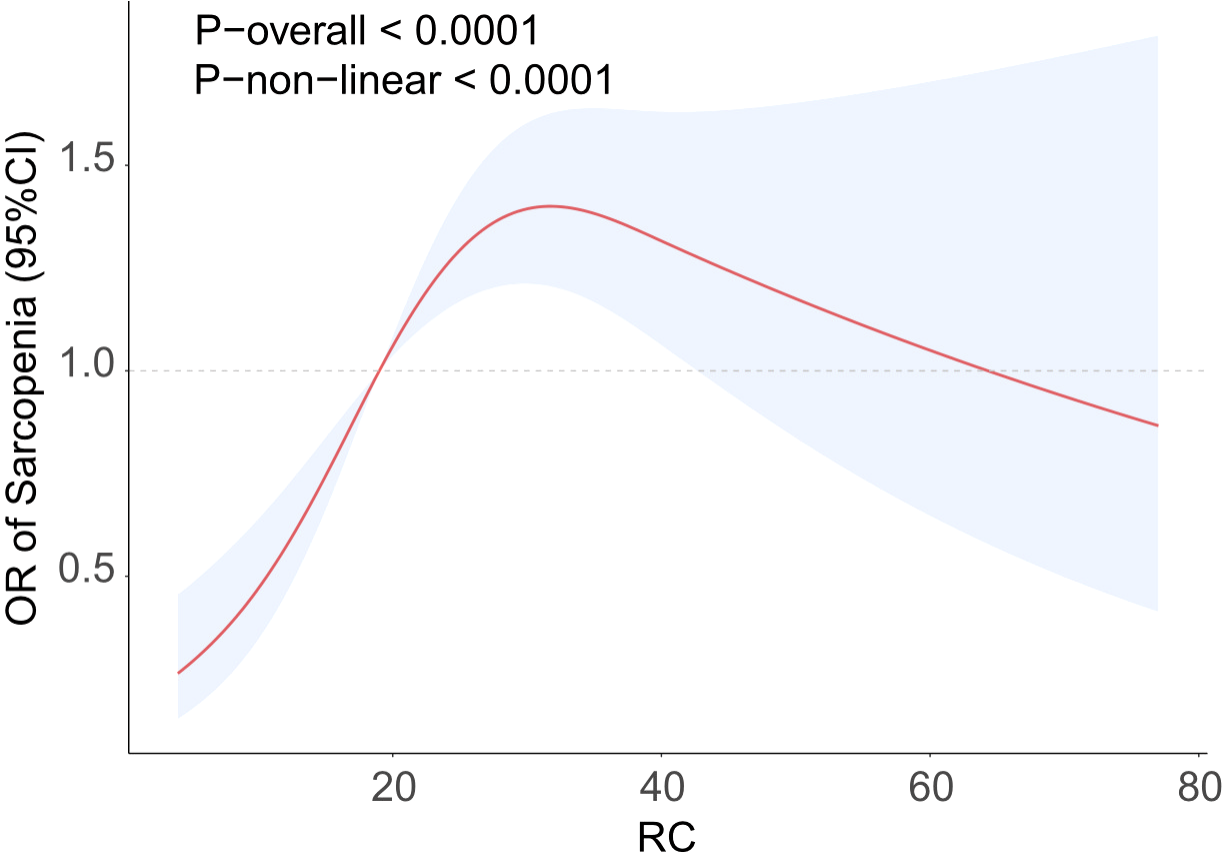
RCS curve fits the Association of RC with Sarcopenia. Adjusted for age, sex, race, educational level, PIR, smoke, activity status, hypertension, hypercholesterolemia, CAD, CKD, diabetes, energy, protein, carbohydrate, total sugars, dietary fiber, total fat.

Analysis using RCS demonstrated a significant nonlinear relationship between RC and the occurrence of sarcopenia (P-nonlinear<0.0001), which took the form of an inverted U-shape (**Figure 2**). The findings demonstrated a positive association between the increase in RC and the progressive increase in the prevalence of sarcopenia, which may eventually plateau after surpassing a certain threshold. An examination of the threshold effect showed that there was a point of inflection at RC=25. **Table 3** presents the segmented logistic regression analysis of the impact on sarcopenia incidence before and after the inflection point. The results indicated that on the left side of the inflection point (RC<25), each unit increase in RC significantly increased the probability of sarcopenia (OR=1.07, 95% CI=1.04-1.10). However, when RC>25, changes in RC had no statistically significant impact on the incidence of sarcopenia (P=0.7).

**Table 3:** Two-stage logistic regression between RC and Sarcopenia.

	RC	OR (95%CI) P-value
Sarcopenia	Standard linear model	1.01 (1.01, 1.02) 0.001
	RC < 25	1.07 (1.04, 1.10) <0.001
	RC > 25	0.99 (0.98, 1.01) 0.300
	Log-likelihood ratio test	<0.001

CI, Confidence Interval; OR, Odds Ratio; RC, Remnant cholesterol.

Adjusted for age, sex, race, educational level, PIR, smoke, activity status, hypertension, hypercholesterolemia, CAD, CKD, diabetes, energy, protein, carbohydrate, total sugars, dietary fiber, total fat.

### 3.4 Subgroup analysis

**Figure 3:**
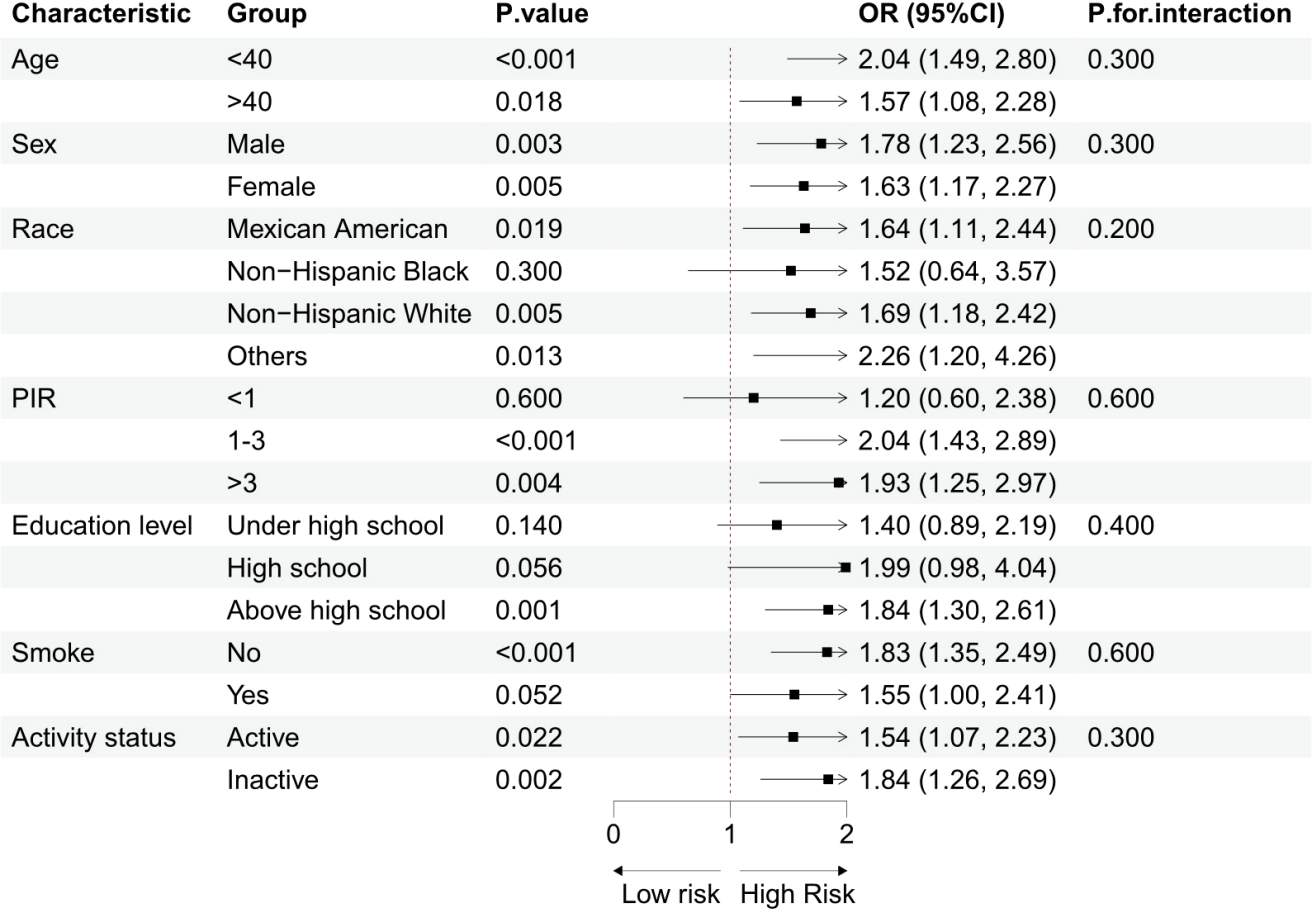
Subgroup analysis of the association between log RC and Sarcopenia.

Subgroup analyses were performed based on demographic and lifestyle factors, and sample size information for each subgroup has been provided in **Table 1**, enabling a clearer assessment of the statistical efficacy of each subgroup. The results, as shown in **Figure 3**, demonstrated that the positive association between log RC and the occurrence of sarcopenia remained consistent across the different groups. The interaction test results indicated no significant interaction between demographic/lifestyle factors and log RC in relation to sarcopenia, supporting the potential of RC as an independent risk factor for sarcopenia.

### 3.5 Sensitivity and supplementary analyses

A sensitivity analysis was conducted by excluding extreme values beyond RC ± 3SD, resulting in a final sample of 4,544 participants, including 4,140 non-sarcopenic participants and 404 sarcopenic participants. After fully adjusting for covariates, the positive association between log RC and sarcopenia remained stable (OR=1.73, 95% CI=1.33-2.24). After converting RC into categorical variables, the results showed that the significant positive association between RC and sarcopenia incidence remained consistent (**Supplementary Table 1**). Additionally, supplementary analyses were conducted to verify the robustness of the study results. **Supplementary Table 2** shows the linear regression analysis results between RC and the SMI, revealing a stable and significant negative association between them. **Supplementary Figure 1** illustrates the nonlinear relationship between the RC and the SMI, indicating that the RC has a more significant impact on the SMI within a specific range. These supplementary analysis findings were consistent with the main study results, further supporting the conclusion of a positive association between RC and sarcopenia.

## 4 Discussion

This research used data from the 2011–2018 NHANES to establish a substantial and favorable association between RC and the prevalence of sarcopenia in adults in the United States. The findings revealed a nonlinear association between RC and sarcopenia, with a crucial threshold discovered at RC=25. The findings were further shown to be strong and consistent using sensitivity and additional analysis. These data indicate that monitoring RC levels might be beneficial for promptly detecting and evaluating sarcopenia.

Recently, the connection between lipid metabolism disorders and sarcopenia has garnered increasing attention. Previous research has consistently shown a link between lipid metabolism problems and sarcopenia (23–25). A study revealed that there is a positive association between the TG/HDL ratio and the prevalence of sarcopenia in older males (26). Similarly, a higher TG/HDL ratio is negatively correlated with muscle mass in patients with T2DM (27). This research demonstrated that RC is a distinct risk factor for sarcopenia. Subgroup analysis further confirmed the RC-sarcopenia association in different age and sex groups, with this association being significant across all strata, suggesting that RC is a universal indicator for assessing sarcopenia.

This study demonstrates a significant association between RC and sarcopenia, suggesting that lipid metabolism disorders may adversely impact muscle health through a series of intricate molecular pathways. Elevated RC levels have been implicated in the initiation of systemic inflammation and oxidative stress, key processes driving muscle protein degradation and impaired synthesis (28, 29). In particular, triglyceride-rich lipoproteins within RC activate monocytes and macrophages, inducing the release of pro-inflammatory cytokines (e.g., TNF-α and IL-6), which accelerate muscle protein breakdown (30, 31). Additionally, the pro-inflammatory environment induced by elevated RC augments oxidative stress, which increases free radical production, damaging muscle cell membranes and mitochondrial function, and disrupting intracellular calcium homeostasis, thereby exacerbating muscle protein degradation and apoptosis (32, 33). Elevated RC has also been closely linked to insulin resistance, a condition characterized by restricted protein synthesis, mobilization of fatty acids, and intensified lipid oxidation, collectively worsening oxidative damage in muscle cells (34, 35). This ultimately impairs energy supply to muscle cells, increasing risks of fibrosis and apoptosis. Collectively, these mechanisms highlight the non-linear association observed between RC and sarcopenia, where an RC threshold of 25 mg/dL may represent a “saturation point” for the deleterious effects of RC. Below this level, increases in RC substantially enhance inflammation and oxidative stress, whereas above this threshold, compensatory mechanisms may partially mitigate the additional negative effects of RC. Similar nonlinear effects have been reported in other metabolic diseases; specifically, the impact of RC on NAFLD disease progression stabilizes beyond a certain threshold, a finding consistent with our results (36). This study suggests that elevated RC levels may increase the prevalence of sarcopenia, highlighting the potential utility of RC level control in the prevention and treatment of sarcopenia. Existing evidence supports the efficacy of both pharmacological and lifestyle interventions in effectively reducing RC levels (37, 38). Future research should prioritize validating the long-term efficacy of these interventions, thereby strengthening the scientific basis for clinical application of RC monitoring and regulation in sarcopenia management.

This study has several strengths. First, the weighted analysis selected a nationally representative sample, accurately reflecting the U.S. population. Second, subgroup analysis was conducted to explore potential impacts in different populations. Third, sensitivity and supplementary analyses further increased the reliability of the study’s conclusions. Nevertheless, this research is subject to many constraints. The use of a cross-sectional design restricts the ability to establish cause-and-effect relationships between RC and sarcopenia. Second, although this study adjusted the model for various covariates, other potential confounding factors, such as the use of lipid-lowering medications, may still exist. Future studies should incorporate medication data to further assess these effects. In addition, this study used the definition criteria of the OARSI, which, unlike the criteria of the European Working Group on Sarcopenia in Older People (EWGSOP), does not include assessments of muscle strength and physical function. Future research could further validate the broader impact of RC on sarcopenia by incorporating parameters of muscle strength and physical function.

## 5 Conclusion

This study demonstrates a significant positive association between RC levels and the prevalence of sarcopenia in U.S. adults, offering novel evidence that RC may serve as a valuable indicator for sarcopenia assessment.

## Data Availability

Publicly available datasets were analyzed in this study. This data can be found here: https://www.cdc.gov/nchs/nhanes/nhanes_products.htm.
