# Supplementary material for: Association between residual cholesterol and sarcopenia in American adults
Source: Front Endocrinol (Lausanne). 2024 Nov 28;15:1461961. doi: 10.3389/fendo.2024.1461961 (PMC11634615; doi:10.3389/fendo.2024.1461961)
Supplement: Supplementary file 1 [file DataSheet1.pdf]

Supplementary Table 1 Sensitivity analysis between RC and Sarcopenia.

		Model 1	Model 2	Model 3
		OR (95%CI) P-value	OR (95%CI) P-value	OR (95%CI) P-value
Sarcopenia	log RC	2.49 (2.01, 3.09) <0.001	2.16 (1.74, 2.68) <0.001	1.73 (1.33, 2.24) <0.001
	Q1	[Reference]	[Reference]	[Reference]
	Q2	2.54 (1.54, 4.20) <0.001	2.28 (1.38, 3.76) 0.002	2.00 (1.13, 3.53) 0.019
	Q3	4.26 (2.96, 6.13) <0.001	3.50 (2.42, 5.05) <0.001	2.95 (1.80, 4.83) <0.001
	Q4	4.61 (3.10, 6.83) <0.001	3.58 (2.44, 5.26) <0.001	2.50 (1.54, 4.05) <0.001
	P for trend	<0.001	<0.001	0.007

CI: confidence Interval; OR: odds ratio; Q: quartiles; RC: remnant cholesterol

Model 1: no covariates adjusted; Model 2: adjusted for age, sex, and race; Model 3: adjusted for age, sex, race, educational level, PIR, smoke, activity status, hypertension, hypercholesterolemia, CAD, CKD, diabetes, energy, protein, carbohydrate, total sugars, dietary fiber, total fat.

Supplementary Table 2 Additional analysis between RC and SMI.

		Model 1<br>β (95%CI) P-value	Model 2<br>β (95%CI) P-value	Model 3<br>β (95%CI) P-value
SMI	log RC	-0.02 (-0.03, 0.00<br>(0.020	-0.05 (-0.06, -0.04)<br><0.001	-0.04 (-0.05, -0.03)<br><0.001
	Q1	[Reference]	[Reference]	[Reference]
	Q2	-0.03 (-0.05, -0.01)<br>0.011	-0.04 (-0.05, -0.03)<br><0.001	-0.03 (-0.05, -0.02)<br><0.001
	Q3	-0.05 (-0.07, -0.02)<br><0.001	-0.06 (-0.08, -0.05)<br><0.001	-0.05 (-0.06, -0.03)<br><0.001
	Q4	-0.02 (-0.04, 0.00)<br>0.036	-0.08 (-0.09, -0.06)<br><0.001	-0.06 (-0.08, -0.05)<br><0.001
	P for trend	0.100	<0.001	<0.001

CI: confidence Interval; OR: odds ratio; Q: quartiles; RC: remnant cholesterol

Model 1: no covariates adjusted; Model 2: adjusted for age, sex, and race; Model 3: adjusted for age, sex, race, educational level, PIR, smoke, activity status, hypertension, hypercholesterolemia, CAD, CKD, diabetes, energy, protein, carbohydrate, total sugars, dietary fiber, total fat.

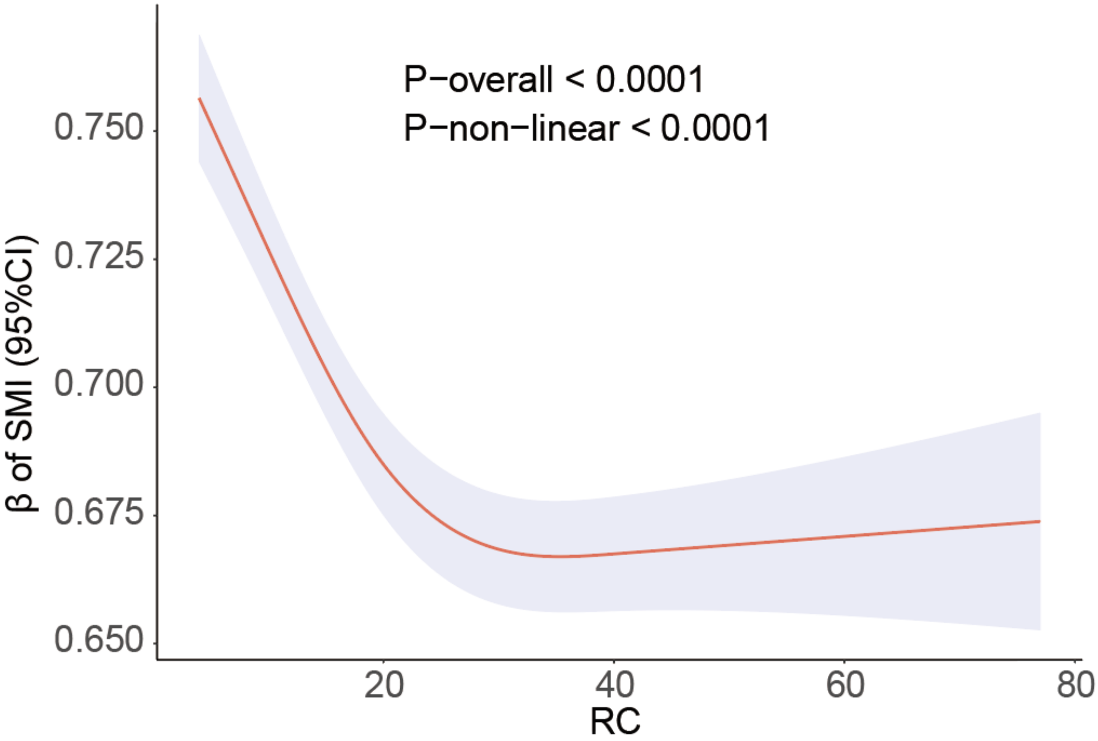

Supplementary Figure 1 RCS curve fits the Association of RC with SMI.

Adjusted for age, sex, race, educational level, PIR, smoke, activity status, hypertension, hypercholesterolemia, CAD, CKD, diabetes, energy, protein, carbohydrate, total sugars, dietary fiber, total fat.
